# Baccalaureate nursing education institutions’ key performance indicators: a review of the existing indicators and qualitative analysis of expert interviews

**DOI:** 10.1186/s12912-023-01484-6

**Published:** 2023-10-05

**Authors:** Marjan Ghofrani, Leila Valizadeh, Vahid Zamanzadeh, Akram Ghahramanian, Ali Janati, Fariba Taleghani

**Affiliations:** 1 Department of Pediatric Nursing, Faculty of Nursing and Midwifery, Tabriz University of Medical Sciences, South Shariati Street, Tabriz, Iran; 2 Department of Medical-Surgical Nursing, Faculty of Nursing and Midwifery, Shahid Beheshti University of Medical Sciences, Tehran, Iran; 3 Department of Medical-Surgical Nursing, Faculty of Nursing and Midwifery, Tabriz University of Medical Sciences, Tabriz, Iran; 4 Department of Health Service Management, Faculty of Management and Medical Informatics, Tabriz University of Medical Sciences, Tabriz, Iran; 5 Nursing & Midwifery Care Research Center, Faculty of Nursing & Midwifery, Isfahan University of Medical Sciences, Isfahan, Iran

**Keywords:** Education, Nursing, Baccalaureate, Quality Improvement, Key performance indicators

## Abstract

**Background:**

Improvements in nurses’ and midwives’ education will increase workforce quantity and quality. Continual improvement of organization performance is based on continual measuring; Appropriate indicators must be selected to measure and appraise the performances. This study aimed to recognize and categorize key performance indicators (KPIs) for baccalaureate nursing education institutions’ performance measurement.

**Methods:**

This study had two phases: (1) Interview: Seven semi-structured interviews were conducted with faculty members and nurses. (2) Narrative Literature review: schools’ and universities’ reports and web pages were assessed to recognize KPIs. The data analysis method was directed content analysis. The Donabedian Model components were used to guide the data analysis.

**Results:**

The total number of indicators retrieved was 468; 75 were from interviews with faculty members and nurses and 393 were from the literature review. Indicators were categorized into: Structure (staff; equipment, resources, and facilities; guidelines), Process (education; communication and collaboration; evaluation), and Outcome (survey and accreditation; national and international recognition; satisfaction; sustainability and financial efficiency; students; alumni; knowledge related to the field).

**Conclusions:**

A number of indicators were identified that were categorized into various groups related to the performance of nursing schools. Further investigations are needed with different groups of stakeholders including students, professional associations, healthcare institutions, alumni, and clients. As well in some areas, new or composite indicators may need to be developed. Also, each institution needs to select appropriate indicators based on its context, policy goals, and infrastructure.

**Supplementary Information:**

The online version contains supplementary material available at 10.1186/s12912-023-01484-6.

## Introduction

Health systems are highly labor-intensive [1], adequate quantity, diversity, and distribution of trained health workers will improve the health system’s capability during a health emergency [2]. Availability, accessibility, acceptability, and quality of the health workforce improve health service coverage and actualizes the highest attainable standard of health [3].

Nurses are the largest group of healthcare workers [4] and play a vital role in the health systems [5]. A sufficient number of qualified health workers needs to be produced and recruited to achieve universal health coverage [6]. Nurses and midwives must also be trained to conduct relevant research and translate research findings into practice to improve best practices and health outcomes [7]. Improvements in nurses’ and midwives’ education will increase workforce numbers and the quality of health care and health systems [8]. Nursing education aims to foster professional nurses who can provide quality care [9]. And strategies focused on high-quality health professions education are necessary to be developed [10]. Competent educators must develop and implement evidence-based curricula that comply with local needs [7].

Countries experiencing war or natural/manmade disasters, those hosting refugees, and those with climate change vulnerability will face specific health workforce challenges that should be considered and addressed [3]. Many disasters like droughts, floods, forest fires, sandstorms, and earthquakes constantly occur in Iran [11]. Also, climate change is severe in Middle East countries, especially Iran [12]. Iran’s constant environmental change in recent years is an issue that cannot be ignored [13]. Besides these, Iran hosts millions of refugees [14]. Currently, 800,000 refugees live in Iran. There are also 2.6 million undocumented Afghans and 600,000 refugees with Afghan passports. Following the upheaval in Afghanistan in 2021, Iran has also welcomed hundreds of thousands of asylum-seekers [15]. The health workforce plays a crucial role in making communities and health systems resilient in responding to natural or man-made disasters and related environmental, technological, and biological hazards and risks [3].

So strengthening nursing and midwifery education is essential [7]. There is a notion that performance measurement systems can improve the quality of services [16]. Key performance indicators (KPIs) provide statistics that enable benchmarking, show how public stakeholders are satisfied with the institution, and how education goals have been met. Key Performance Indicators that are well-selected and measurable can specify which parts must take specific actions, and the quality can be improved [17].

Reporting nursing education performance indicators in Iran as a developing country with the specific challenges that were mentioned has been neglected. Almost none of the nursing schools have a management dashboard that can provide a proper performance report to the different groups of stakeholders. The use of performance indicators seems necessary for all nursing schools. Many nursing schools around the world use them. We decided to design a set of indicators for this goal. One of the first steps in designing a set of indicators is to identify the existing indicators and consult with stakeholders. We think that the publication of the results of this study can be of help to other nursing experts around the world who intend to design performance indicators for their institutions. We think the findings of this study can offer some possible indicators in an organized accessible way that would help nursing experts to use them after more validation, proper modification, and gaining consensus based on their aims and target population for the indicator set.

Considering these, in this study, we aimed to recognize and categorize key performance indicators for baccalaureate nursing education institutions’ performance measurement.

## Materials and methods

This study has two phases: interview with stakeholders, and a literature review on existing KPIs.

### Interview phase

#### Design

The design of this phase of the study is exploratory-descriptive qualitative. The main purpose of exploratory-descriptive studies is to describe or explore a phenomenon, problem, or subject and includes a wide range of questions on individuals’ experiences, knowledge, attitudes, emotions, and perceptions, or views points [18].

#### Sampling

Purposive sampling was used. This technique is used to reach people who can provide us with rich information [19]. We continued the interviews until data saturation was achieved. Saturation is the criterion for discontinuing data collection and/or analysis and the gold standard for determining purposive sample sizes in health science research [20]. We had seven participants in this phase.

#### Participants

Decision-makers have come to understand the importance of stakeholder engagement and public participation for effective decision-making and greater public acceptance [21]. Stakeholders in health care education are classified as internal or external to the academic organization. Internal stakeholders include program faculty and students. External stakeholders include professional associations, health care institutions, alumni, and clients [22].

Inclusion criteria: In this study, the target stakeholders were faculty members (internal stakeholders) or nurses (external stakeholders) involved in policymaking in nursing, professional associations members, or have experience in a hospital or university management.

We intended to choose people who can provide us with the best information, we invited academic staff members or nurses to participate in the study who can provide us with information in various fields of nursing education institutions performance. For this reason, the participating members had a variety of experiences related to the field. The only criteria for entering the study:

- Nursing graduate.
- Engaging in nursing related activities and job positions.

And the only exclusion criterion:

- Reluctance to participate in the study.

The participants’ experiences included: Ministry of health membership, faculty members, school/college management, hospital management, world health organization regional experts network membership, national nursing research network membership, professional and scientific associations membership, insurance officer, accreditation committee membership, scientific journals’ editor in chief, scientific journal editorial team, clinical experiences, national nursing policy council membership, nursing board membership, school’s education development office membership, and sports championships. The mean years of work experience were 22 years. Five participants had Ph.D., and two participants had M.Sc. in nursing. One of the participants was professor, and two were associate professors.

#### Setting

Participants worked in nursing and midwifery schools, the ministry of health, and Tabriz University of Medical Sciences’ management office. Nursing and midwifery schools are affiliated with Tabriz University of Medical Sciences, Tarbiat Modares University and Bushehr University of Medical Sciences. Based on Webometrics these universities’ world ranks are 857, 1565, and 2904 respectively [23]. The baccalaureate nursing curriculum is the same all over the country. Universities and the health ministry are located in different cities, from the capital to border states. The cost of living, income, and even common speaking language among local people is other in these cities. Towns’ climate, environmental status, and population density are also entirely different. All three universities have dormitories, accept students from all over the country, and have fee-paying and free-paying students.

#### Data collection

We conducted semi-structured interviews with stakeholders. Interviews lasted between 28 and 60 min. We had one main open-ended question and some other exploring questions in case of necessity to explore more about the topic. The main question was:

Which performance indicators should be used to measure a nursing education institution’s performance related to baccalaureate nursing education?

Sessions were recorded, and further investigation was conducted on the data.

### Literature review phase

We searched to recognize key performance indicators (KPIs)/performance indicators (PIs) that universities worldwide had reported. We found 30 reports and web pages. Based on inclusion criteria, 20 were included in the data analysis (available as supplementary material). Based on Grant & Booth: “A literature review involves some process for identifying materials for potential inclusion—whether or not requiring a formal literature search—for selecting included materials, for synthesizing them in textual, tabular or graphical form and for making some analysis of their contribution or value” [24].

#### Inclusion criteria for indicators

(1) Reported specifically as KPIs/PIs, (2) Reported by nursing schools/colleges or universities that have nursing schools/colleges, (3) Reports containing these indicators had to be published after 2015. (4) The most recent available version of the report had to be considered. (5) Indicators must be related to baccalaureate nursing education.

#### Search strategy

Search conducted between April and May 2021, we also reconsidered the webpages in April 2022, as they may be updated. We continued the search until most of the indicators were repeated. The search languages were limited to English and Persian. We used “performance measurement”, “performance management”, “KPI”, “key performance indicators”, “performance indicators”, “PI”, “nursing school”, “nursing education”, and “nursing education institutions” as keywords. Indicators were retrieved and saved in a file until further investigation was placed on them.

### Data analysis and integration

Audio-recorded interviews were transcribed verbatim by the first author. These transcripts along with the file prepared from the literature review were analyzed. Directed Content analysis was used for data analysis. Content analysis is designed to classify the words in a text into categories [19]. With a directed qualitative content analysis a theory or theoretical framework would be validated, refined and/or extended in a new context [25]. We guided the data analysis in this study with the use of the Donabedian Model which is the most recognized and comprehensive quality evaluation framework [26]. This model has three components, structure, process and outcome [27]. Donabedian Model measures the differences between expected and actual performance, to identify gaps that can be the first step in quality improvement [28]. Using this Model will help us to understand what has led to outcome, not just what is outcome [27]. This Model is widely used including several studies in the educational context [29, 30].

Based on this definition, we consider structure as all the attributes in and with nursing education occur, process as all the acts of nursing education, and outcome as all the effects of nursing education.

**Fig. 1:**
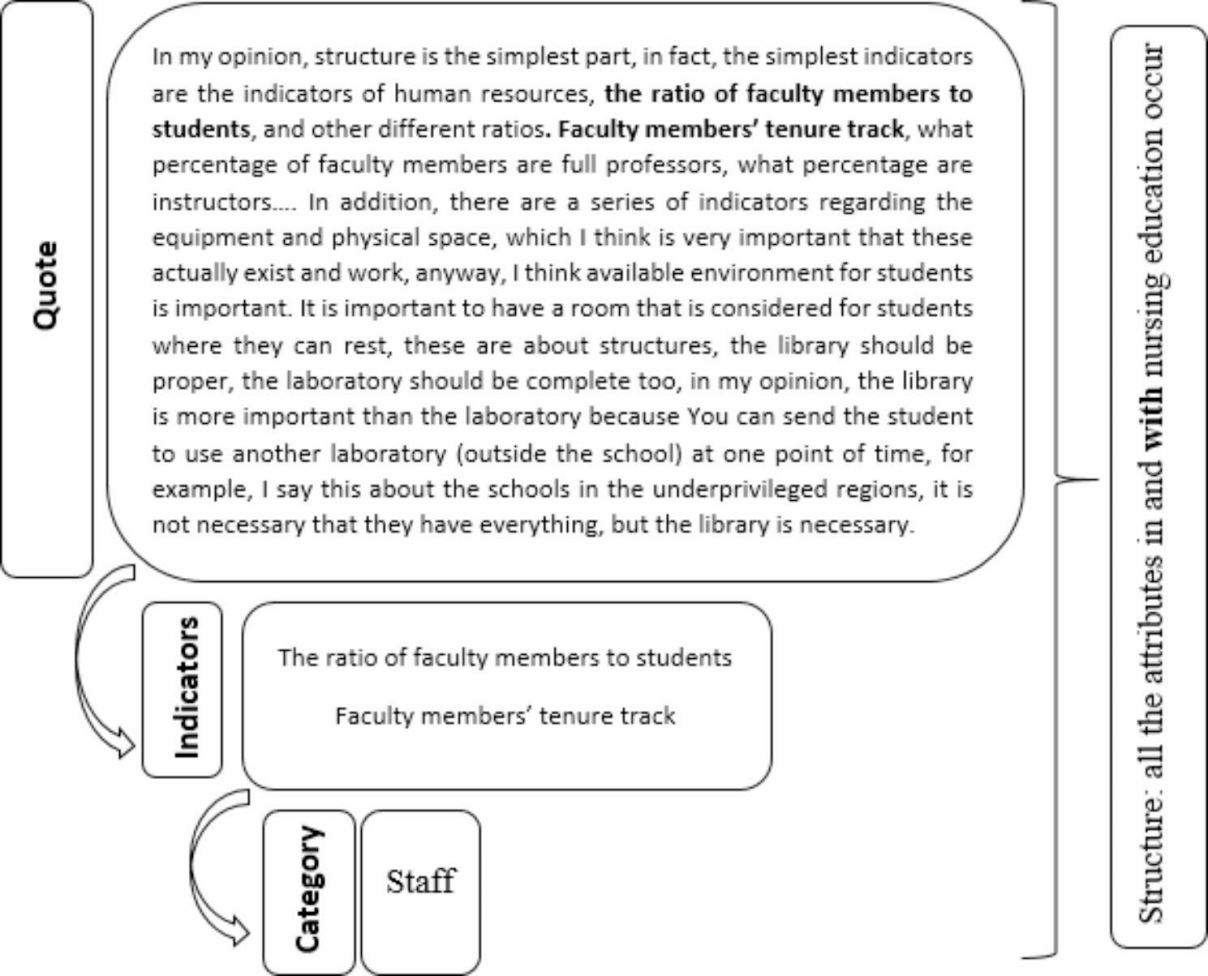
An example of directed content analysis process. Finding and categorizing indicators and assigning them to the components of the Donabedian Model

The data analysis process involved extracting the performance indicators and categorizing them, and assigning the categories to 3 components of the Donabedian Model (Fig. 1). Repeated indicators were omitted, and the most comprehensive one was selected between similar ones. During the study group discussions, indicators were also assessed to be (1) Measurable, or (2) Not specific to a culture, region/country, or university. Each study group discussion session included four of the authors.

The study group members have experiences in nursing and midwifery school management, national nursing research network management, membership in the board of nursing, membership in nursing and midwifery evaluation committees, hospital management, membership in scientific journals editorial offices, membership in hospital and schools’ accreditation bodies, and clinical experiences.

One of the authors with relevant expertise also rechecked indicators related to financial issues. Indicators from interviews and literature review were integrated during the data analysis process.

### Trustworthiness

Interviews were conducted by MG and transcribed verbatim by herself. This will reduce the possibility of mistakes and misunderstandings in transcribing the interviews. To improve the study’s rigor, LV reviewed and checked the data collection processes. Assessment, choosing, and categorizing the indicators took place in a group of four authors (MG, LV, VZ, and AG) with relevant expertise (investigator triangulation). Some of the categorizations were later rechecked with another author (AJ) (peer debriefing). Final findings were also checked with one of the participants too (member check).

### Ethical considerations

The ethics committee of Tabriz University of Medical Sciences approved this study (IR.TBZMED.REC.1399.1128**)**. Confidentiality and anonymity for the research study participants were provided. The aim of the research and the participants’ role was explained, and written informed consent was taken from all the participants. Permission was obtained to audio record the sessions.

## Findings

The total number of indicators retrieved was 468; 75 were from interviews and 393 from the literature review. The indicators obtained from the literature review are available as supplementary materials. After duplicate removal, assessment, and integration, 112 indicators were categorized into 13 groups. Later these categories were assigned to three components of the Donabedian model (Table 1).

**Table 1:** Selected indicators, their categories, and category assignment to the Donabedian Model’s components

Component	Category	Indicators
	1. Staff	1. Ratio of students to administrators’ staff. 2. Ratio of students to teaching staff. 3. Staff by qualification and gender. 4. Tenure track faculty headcount. 5. Indigenous staff participation rate. 6. The proportion of faculty members with more than two years of clinical experience. 7. The proportion of faculty members with expertise in clinical management. 8. The proportion of faculty members with executive experience outside of college. 9. The proportion of teaching staff participating in professional development activities. 10. Staff turnover.
	2. Equipment, resources, and facilities	1. The average number of computers available for each student’s use. 2. The number of databases retina available to students and faculty members through the library. 3. The quality of the digital library. 4. The number of online full-text dissertations. 5. Books ratio to students. 6. The overall quality of the facilities/resources in the college. 7. The overall quality of the services in the college. 8. The number of active strategic partners, including schools trusts and hospitals. 9. Buildings, Grounds, Land, Academic and Administrative Space per full-time equivalent. 10. Total grants received. 11. Institutional scholarship total amount. 12. Total annual donor commitments. 13. The income ratio that comes from different resources. 14. Annual budget. 15. Funding.
**Structure**	3. Guidelines	1. The extent of awareness of the department teaching staff, the students, and the administrative staff of the existence of general regulations and policies concerning and affecting them. 2. Stakeholder evaluation of the Policy Handbook, including administrative flow chart and job responsibilities.
	4. Education	1. Teaching effectiveness-student evaluation. 2. Teaching effectiveness-chairman evaluation. 3. Teaching effectiveness-peer evaluation. 4. Students’ overall rating on the quality of their courses. 5. Students’ evaluation of the value and quality of field activities. 6. The appropriateness of the teaching methods according to student evaluation, external reviewers, and the teaching staff for each of the learning domains. 7. The appropriateness of the qualifications and experience of the teaching staff for the courses they teach. 8. Quality of online teaching. 9. Online Student Credit Hour. 10. The volume of continuing professional development work delivered.
**Process**	5. Communication and collaboration	1. Level of student engagement. 2. Weekly Student Contact Hours. 3. The number of periodic and regular meetings between faculty members, staff, and managers. 4. Membership in international organizations. 5. International activities by faculty members per college. 6. The number of international research collaborations. 7. Number of faculty engaged in professional organizations. 8. The number of membership of boards and committees outside the University. 9. The number of knowledge transfer partnerships and other enterprise partnerships with external businesses. 10. The proportion of full-time teaching and other staff actively engaged in community service activities. 11. High impact practices participation.
	6. Evaluation	1. Stakeholder evaluation of Overall: (a) Websites, (b) e-learning services, (c) Hardware and software, (d) Accessibility (e) Learning and Teaching, (f) Assessment and service, (g) Web-based electronic data management system or electronic resources. 2. The evaluation of the quality and usefulness of the courses by advisory bodies of the industrial and professional sectors and other distinctive community sectors. 3. Evaluating the effectiveness of governance and leadership. 4. Evaluating the general performance of administration. 5. Student evaluation of academic and career counseling.
**Outcome**	7. Survey and accreditation	1. Campus climate survey. 2. National student survey and internal survey ratings. 3. Outcome of alumni survey. 4. The outcome of staff survey. 5. Evidence of current accreditation by a national nursing accrediting body.
	8. National and international recognition	1. Number of faculty/student awards granted. 2. School international ranking. 3. School national ranking. 4. Total webpage visits along with new visitors. 5. Top-of-Mind Awareness. 6. Web Analytics. 7. Earned Media.
	9. Satisfaction	1. Student satisfaction rate. 2. Graduate satisfaction rate. 3. Staff satisfaction rate. 4. Patient satisfaction rate. 5. Patients’ family satisfaction rate. 6. Employer Satisfaction rate.
	10. Sustainability and Financial Efficiency	1. Energy Use Index. 2. Carbon neutrality. 3. The Association for the Advancement of Sustainability in Higher Education Evidence. 4. Composite Financial Index. 5. Student Loan Repayment Rate. 6. Annual cost per student. 7. Total teaching & learning expenditure. 8. Total operating expenditure. 9. The student’s services financial stake according to the total operational expenses. 10. Total expense of the university on the research. 11. Faculty Instructional Full Time Equivalent paid in program.
	11. Students	1. Enrollment rate. 2. Student entry tariffs score. 3. The students’ cumulative grade point average (CGPA). 4. Health Education Systems Incorporated (HESI) exit examination. 5. National Educational Examinations passing rates. 6. Average number of semesters a student spends under probation. 7. College-level course success rate. 8. Total number of online degrees conferred. 9. Total bachelor’s degrees conferred. 10. Transfer-in rate. 11. Transfer-out rate. 12. Retention rate. 13. Graduation rates. 14. On-Time graduation rate. 15. Assessment of Student Learning: student exit survey. 16. Assessment of Student Learning: alumni and employers’ surveys.
	12. Alumni	1. Level of Interest in the field 2. Graduates assessment results (Cognitive, Function, Attitude, Problem Solving, communication, decision making, management, planning, familiarity with legal issues, social responsibility, altruism, self-confidence, professionalization, professional dignity, and ethics). 3. Proportion of graduates from undergraduate program who enrolled in further study. 4. Passing Rates for Licensure Exams (like NCLEX-RN). 5. Employment rate. 6. Percentage of immigration and finding related jobs abroad. 7. Quality of provided care by alumni. 8. Nurse-sensitive indicators.
	13. Knowledge related to the field	1. The number of publications in the form of books, book chapters, and technical reports. 2. The number of scientific papers presented in the conferences on the national or international level. 3. The number of research and innovations registered as intellectual property and patents. 4. The number of scientific researches published in the scientific refereed journals. 5. The number of citations in scientific refereed journals of all teaching staff members’ research 6. The number of nursing models developed.

Donabedian hypothesized that there are connections between structure, process, and outcome. Good structure should promote good process, which leads to good outcome (unidirectional pathway) [31]. Based on the definition of components, and hypothesized connections between them, categories were assigned to components.

**Fig. 2:**
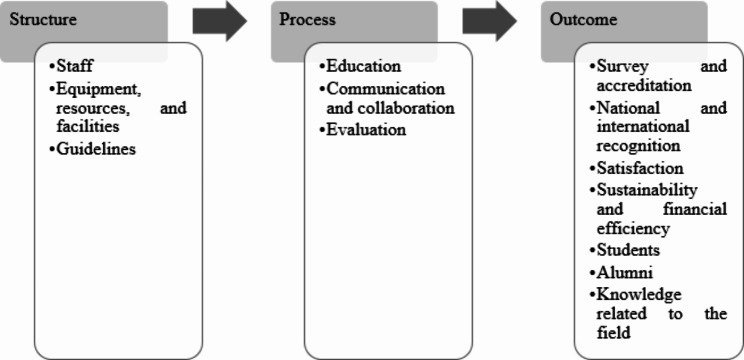
Donabedian’s three components model for nursing education based on interviews and literatures reviews

The structure component had three, the process component three, and the outcome component seven categories (Fig. 2).

## Discussion

This study aimed to recognize and categorize key performance indicators for baccalaureate nursing education institutions’ performance measurement. During the review, interviews, and data analysis process, eventually, 112 indicators were categorized into 13 groups: structure (staff; equipment, resources, and facilities; guidelines), process (education; communication and collaboration; evaluation), and outcome (survey and accreditation; national and international recognition; satisfaction; sustainability and financial efficiency; students; alumni; knowledge related to the field).

This study categorized indicators into three groups (structure, process, outcome). Based on the Donabedian quality improvement model, we assumed that good structures would affect the process, and the good process will lead to expected outcomes. There is a unidirectional connection between these three components. Proper measurement of these components in nursing education will reveal weak points. And eventually may help us to improve nursing education institutions’ performance.

Different university activities, including educational, scientific, international, and financial, should be considered in an indicator system. Selected indicators should reflect university interaction with industries and geographical regions, the policies regarding academic entrepreneurial initiatives, knowledge, research and technologies transfer, participation in international scientific research networks, and collaborations [32]. Their functions also include elements such as administration, curriculum, learning outcomes, faculty efficiency, and resource allocation [33].

In the current study, we reported several indicators related to these topics placed in the structure, process, and outcome components. Categories such as “Staff”, “Equipment, resources, and facilities”, “Communication and collaboration”, “National and international recognition”, “Students”, and “Knowledge related to the field” contain indicators that represent aspects of these topics. As data on educational, scientific, and research, international, financial, and economic performance, and international public recognition of Russian universities were assessed by key performance indicators in a study by Guseva et al. [32].

Environmental performance is progressively essential to organizational decision-making boards [34]. The best worldwide higher education institutions have implemented environmental management systems and taken part in campus sustainability to take action to reduce or prevent climate change by reducing air pollution from transportation, and waste from campus facilities, increasing research-and-development potential toward green campus initiatives [35]. Lo-Iacono-Ferreira et al. offered a list of over 140 environmental indicators and defined seven Key Performance Indicators, three of which were thoroughly assessed. Energy consumption, waste management treatment, and greenhouse gas emissions are the key elements of these three indicators [34]. In our study, except for a few indicators, we didn’t find indicators related to the environmental aspect of sustainability. Also, during interviews, participants didn’t mention an indicator that measures school performance related to this concept. Considering the increasing importance of sustainability in higher education institution performance, it is clear that there is a need to attract all the people’s attention toward this issue, especially those involved in higher education management and education.

In a study by Lazic et al. aimed to propose a quality assessment model for higher education institutions in the technical–technological field and a system for decision support and optimal management strategies for quality improvement, a set of indicators was presented and divided into six groups or dimensions: institution, teaching, science, service users (parents, students), employers/economy, country/Society. The stakeholders recognized the following indicators as the most influential indicator in these six groups: an average grade from a previous educational level, number of study programs, academic staff mobility, the average duration of studies, projects with business entities, public lectures [17]. Findings of this study is also compatible with our research.

Present study’s findings may be some help for those nursing managers, nursing policy makers or any other related person whose intended to develop a set of performance indicators for their institution. Also each country or nursing education institution needs to select appropriate indicators based on its context, policy goals, and available infra-structures, but indicators presented in this study can be considered as a recommendation and be used after proper consideration and further discussion.

In this study, we selected our participants based on their experiences, positions, activities, and backgrounds in nursing, and of course, their accessibility and willingness to participate in the study. The people who participated in the interviews are working in three different universities, which are among the first-rate or average universities in our country. The presence of participants from universities with higher rankings in the world could have provided us with different data, although we could not access them during the present study, which could be one of the limitations of our study. In addition, considering other groups of stakeholders’ viewpoints may provide broader perspective of the nursing education expected performance. Also it can be useful if a review takes place on sources other than nursing schools’ reports or webpages, as they may have introduced other indicators that are new and applicable in nursing education performance measurement.

Using multiple sources of data collection can be considered as a strength for presents study’s findings as this have involved various researchers’ opinion thought out the literature review and interviews. Also the efforts taken place to improve the trustworthiness can make the findings more reliable. We also provided a supplementary file consisted of all the indicators that were retrieved during literature review, this file may help other researchers to assess and decide how much these findings are reliable.

## Conclusions

Although we believe this study’s finding isn’t complete- because there may be more indicators that aren’t recognized by the researchers in the present study, other nurse experts may have got other suggestions, and some more indicators is even needed to be developed- but we think it can be some help to select nursing education KPIs and design a management dashboard for nursing schools/colleges. Further investigation is required to develop other possible indicators, especially in more emerging topics. Selected indicators in schools and colleges must provide accurate information to benchmark institution performance against other institutions, its past performance, or standards. Each institution needs to select appropriate indicators based on its context, policy goals, and infrastructure. Findings of this study shows different categories of indicators related to structure, process, and outcome. But the outcome component got the most categories, this may be due to the importance of assessing this aspect of school performance.

### Electronic supplementary material

Below is the link to the electronic supplementary material.

Supplementary Material 1

Supplementary Material 2

## Data Availability

The datasets used and/or analyzed during the current study are available from the corresponding author on reasonable request.

## Abbreviations

KPIs: Key performance indicators
PIs: Performance indicators
