# Supplementary material for: Baccalaureate nursing education institutions’ key performance indicators: a review of the existing indicators and qualitative analysis of expert interviews
Source: BMC Nurs. 2023 Oct 5;22:357. doi: 10.1186/s12912-023-01484-6 (PMC10552274; doi:10.1186/s12912-023-01484-6)
Supplement: Supplementary file 1 — Supplementary Material 1 [file 12912_2023_1484_MOESM1_ESM.docx]

**Indicators that were retrieved from the literature review phase:**

1. **Graduation Rate**
2. **Employment Rate**
3. **The 2016 default rates** (loans) ([1](#_ENREF_1)).
4. **Graduate Satisfaction**
5. **Student Satisfaction**
6. **Employer Satisfaction**
7. **The Employment Rate**
8. **The Graduation Rate** ([2](#_ENREF_2)).
9. **KPI#1:** Steps to revitalize curriculum are within the timeline established by the School of Nursing
10. **KPI#2:** 80% of graduates from both programs (Associate of Applied Science and Bachelor of Science in Nursing) averaged over the most recent three-year calendar time period will pass NCLEX-RN on the first attempt
11. **KPI#3:** 100% of full-time nursing faculty will attend professional development as long as the budget allows
12. **KPI#4:** 100% of nursing faculty/clinical instructor positions will be filled with qualified faculty.
13. **KPI#5**: 80% of newly admitted Associate of Applied Science students will graduate from the program within one year from admission into the nursing sequence
14. **KPI#6:** 60% of Bachelor of Science students admitted will graduate from the Bachelor of Science program within three years from admission into the nursing sequence.
15. **KPI#7:** A School of Nursing representative will attend at least three high school recruitment events and make a visit at surrounding technical nursing programs for recruitment into the school of nursing programs ([3](#_ENREF_3)).
16. **Expenditures**
17. **Tenure track Faculty Headcount**
18. **Faculty Instructional Full Time Equivalent:** For FY03 forward, the actual FTE in faculty positions paid in program 01, state funds (as of November payroll).
19. **TT & NT Instructional Full Time Equivalent (FTE) and % Tenure-track:** For FY03 forward, the actual FTE in faculty positions holding ranks of Assistant, Associate, or Full Professor, or with tenure status of continuous tenure, paid in program 01, state funds (as of November payroll). Prior to FY03, this was budgeted FTE in lines with rank.
20. **GTA FTE:** For FY03 forward, actual headcount graduate assistants (GTA, GRA, and GSA positions) with any portion of their stipend paid in program 01, state funds (as of November payroll), divided by 2. All GTAs are assumed to be .5 FTE for this report. Prior to FY03, this was budgeted FTE in GTA lines.
21. **SCH Lower, Upper, Grad, and Total:** Starting in FY05, student credit hours produced by faculty in the department, in summer, fall, and Spring terms, as of census date each term. Graduate students taking 400 level courses are counted in the graduate SCH row. Credits follow tenure track faculty to their home departments or are split across departments if the appointment is split. For example, a course taught by a tenure-track faculty member in Ecology in the BIOL subject code is counted in Ecology. A CLS 101US course taught by a tenure-track faculty member in Electrical and Computer Engineering is counted in ECE. Credits taught by a faculty member with a 30/70 split appointment are split 30/70 regardless of course subject code. Credits taught by adjunct faculty are counted in the department associated with the subject code. These numbers are similar but not identical to those generated in the Provost's Reallocation Model and the Delaware Study of Instructional Costs and Productivity. Prior to FY05, most credit hours were counted in the department associated with the subject code, with some exceptions (notably BIOL and University Seminars), where faculty affiliation was followed.
22. **Online Student Credit Hour**
23. **% Taught by Ten Track/SCH Lower:** Percent of lower division student credit hours counted in the department that are taught by tenure-track faculty in the department. This includes university seminar instruction by tenure-track faculty. **% Taught by Ten Track/Sections Lower:** Percent of lower division sections counted in the department that are taught by tenure-track faculty in the department. This includes university seminar instruction by tenure-track faculty. In 2001, recitations were considered labs and not included; after 2001, credit-bearing recitations were included.
24. **Total SCH/Faculty FTE and Total SCH/Faculty FTE w/GTAs:** The ratio of total FY SCH to Faculty FTE, without and with GTA FTE, as reported above
25. **Undergrad, Grad, Total FY FTE:** Student FTE based on SCH reported above according to Montana University System definitions. Undergraduate FTE = 30 SCH per year. Graduate FTE = 24 SCH per year. Graduate students taking 400 level courses are counted as graduate FTE.
26. **Expend/Student FTE:** The ratio of expenditures to total FY FTE as reported above.
27. **Student FTE/Faculty FTE and Student FTE/Faculty FTE w/ GTAs:** The ratio of total student FTE to faculty FTE without and with GTA FTE, as reported above. **Majors:** Headcount of majors in the department, by class standing, as of the census date of the Fall semester.
28. **Majors/Faculty FTE:** ratio of all majors, including second majors, to faculty FTE (excluding GTAs), as reported above.
29. **Degrees awarded** ([4](#_ENREF_4))
30. **Evaluation of Educational Experience quality averages by questioning the senior students.**
31. **The percentage of the courses which was evaluated by students during the educational year.**
32. **The average of whole student evaluation of program courses based on 5 points scale.**
33. **The percentage of students that finish course requirements in the minimum allowed period of time.**
34. **The average of student's opinions regarding academic and professional**
35. **Counseling services. the number of lectures and courses in self-development sector**
36. **The average of opinions of the working sectors in the skills of graduated students**
37. **The percentage of academic members and participants in professional development activities during the academic year.**
38. **The number of suggestions in specialties to be renew**
39. **The number of new specialties in the program.**
40. **The percentage of accepted students in new specialties.**
41. **The average of working areas opinions in new learning outcomes and the skills of program graduate**
42. **The percentage of those employed in the new applied areas within 6 months after graduation.**
43. **The average of the new graduates in the new learning outcomes**
44. **The number of airtight research during the last year.**
45. **The percentage of the academic members whom has at least one published research during the last year.**
46. **The number of topics derived from academic member's research in courses.**
47. **The number of academic members of the program participating in community service activities** ([5](#_ENREF_5)).
48. **Stakeholders' awareness ratings of the Mission Statement and Objectives**
49. **Stakeholder evaluation of the Policy Handbook, including administrative flow chart and job responsibilities**
50. **Students' overall evaluation on the quality of their learning experiences.**
51. **Proportion of courses in which student evaluations were conducted during the year.**
52. **Proportion of programs in which there was an independent verification, within the institution, of standards of student achievement during the year**
53. **Proportion of programs in which there was an independent verification of standards of student achievement by people (evaluators) external to the institution during the year.**
54. **Ratio of students to teaching staff. (Based on full time equivalents)**
55. **Students overall rating on the quality of their courses. (Average rating of students on a five point scale on overall evaluation of courses.)**
56. **Proportion of teaching staff with verified doctoral qualifications**
57. **Retention Rate**
58. **Graduation Rate for Undergraduate Students**
59. **Proportion of graduates from undergraduate programs who within six months of graduation are: a) employed b) enrolled in further study c) not seeking employment or further study**
60. **Ratio of students to administrative staff**
61. **Proportion of total operating funds (other than accommodation and student allowances) allocated to provision of student services**
62. **Student evaluation of academic and career counselling.**
63. **Stakeholder evaluation of library and media center. (Average overall rating of the adequacy of the library & media center, including:** a) Staff assistance b) Current and up-to-date c) Copy & print facilities d) Functionality of equipment e) Atmosphere or climate for studying f) Availability of study sites, and g) Any other quality indicators of service on a five- point scale of an annual survey.)
64. **Annual expenditure on IT budget, including:** a) Percentage of the total Institution, or College, or Program budget allocated for IT b) Percentage of IT budget allocated per program for institutional or per student for programmatic c) Percentage of IT budget allocated for software licenses; d) Percentage of IT budget allocated for IT security; e) Percentage of IT budge allocated for IT maintenance.
65. **Stakeholder evaluation of the IT services.** (Average overall rating of the adequacy of: a) IT availability, b) Security, c) Maintenance, d) Accessibility e) Support systems, f) Software and up-dates, g) Age of hardware, and h) Other viable indicators of service on a five- point scale of an annual survey.
66. **Stakeholder evaluation of Overall :** a) Websites, b) e-learning services c) Hardware and software d) Accessibility e) Learning and Teaching f) Assessment and service g) Web-based electronic data management system or electronic resources
67. **Total operating expenditure (other than accommodation and student allowances) per student**
68. **Proportion of teaching staff leaving the institution in the past year for reasons other than age retirement.**
69. **Proportion of teaching staff participating in professional development activities during the past year.**
70. **Number of refereed publications in the previous year per full time equivalent teaching staff.**
71. **Number of citations in refereed journals in the previous year per full time equivalent faculty members.**
72. **Proportion of full time member of teaching staff with at least one refereed publication during the previous year.**
73. **Number of papers or reports presented at academic conferences during the past year per full time equivalent faculty members**
74. **Research income from external sources in the past year as a proportion of the number of full time faculty members.**
75. **Proportion of the total, annual operational budget dedicated to research**
76. **Proportion of full time teaching and other staff actively engaged in community service activities.**
77. **Number of community education programs provided as a proportion of the number of departments (**[**6**](#_ENREF_6)**).**
78. **Assessment of Student Learning:** Direct measures such as ATI end-of-course examinations, HESI exit examination, NCLEX-RN Examination. Indirect measures include student exit survey, alumni and employers' surveys.
79. **Number of students enrolled**
80. **Retention and graduation rates**
81. **Number of graduates produced**
82. **Number of students participating in service learning**
83. **Faculty scholarship productivity.**
84. **Teaching Effectiveness** (peer evaluation, student evaluation, chairman evaluation) ([7](#_ENREF_7)).
85. **Employment Rates - University Graduates**
86. **Graduation Rates**
87. **OSAP Loan Default Rates** ([8](#_ENREF_8)).
88. **Student academic performance** This indicator measures the performance of the students through their cumulative grade point average (CGPA)
89. **Academic Probation** The indicator for probation consists of two parts: I. The first part measures the percentage of the number of students on probation relative to the total number of students (college or university wise). II. The second part measures the average of the number of semesters a student spends on probation. (Percentage of students under probation per college and University, Comparison of the percentage of students under probation in the Present and Previous Periods, Average number of semesters a student spends under probation, Comparison of the number of semesters a student spends under probation in the Present and Previous Periods)
90. **Student loss** ( for academic or non-academic reasons) This indicator is the percentage of the number of students who leave the University (for academic or non-academic reasons) before they complete their degree programme to the total number of registered students in the semester.( Student loss at college level, Student loss at University level)
91. **Student transfer from college to college** (Transfer between colleges, Transfer indicator: This transfer indicator is the ratio of dividing the result of subtracting the number of students who transferred out of the college from the number that transferred to the college by the number of students in the college before the transfer. The ratio is presented as a percentage. It can be positive indicating the college has gained in student numbers or negative in which case the college has lost in student numbers.)
92. **Graduation** (Classification of graduation degree per college, Comparison of graduation degree levels of the different colleges, Classification of graduation degrees of the University students, Average CGPA of graduate, Comparison of the average CGPA of graduates of the Present and Previous Periods, The General graduation Indicator: The graduation indicator is a measure of the estimated ratio of the students who graduated that year as compared to the total expected to graduate if every student graduated on time. A graduation indicator of 1.0 means that all the students graduated on time that year. It is calculated for every college separately because it depends on the number of semesters for completing the graduation requirements, Comparison of general graduation indicator of the Present and Previous Periods)
93. **Teaching** (Percentage of sections taught with 30 students or less per college, The average percentage of sections taught with 30 or less in the University, Teaching Load (FTE ) Teaching load of an instructor is the equivalent number of students with full teaching load per instructor, Comparison of the FTE for the Present and Previous Periods
94. **Academic publications** (Papers published in refereed periodicals, Comparison of indicator for refereed publications of the Present and Previous Periods, Papers presented at international conferences, Comparison of indicator for conference presentations in the Present and Previous Periods, Publications in the form of books, book chapters and technical reports, Annual number of published books per college, Comparison of the number of books published during the present and Previous Periods, Annual number of published book chapters, Comparison of book chapters published during the Present and Previous Periods, Annual number of published technical reports,
95. **Conferences** (Attendance at international scientific conferences, Comparison of indicator of international conference attendance during the Present and Previous Periods, International scientific conferences organized by the University, Research projects funded by the University annual budget, Research projects funded by agencies outside university,
96. **Community Service** (Training courses of the Centre for Community Service and Continuing Education (CCSCE), Membership of boards and committees outside University, Media Activities, Participation in public activities outside University,
97. **International Cooperation** (Various agreements with the relevant institutions, Membership in international organizations, General scientific cooperation, International activities by Faculty members per college, International activities by Faculty members at University level, Comparison of indicator for international cooperation of Faculty during the Present and Previous Periods)
98. **University Environment** (Staff satisfaction, Staff satisfaction with services provided, Benefits and sense of job security, Work Environment, General services, Teaching (for faculty members only), Research (for Faculty only), The average satisfaction of staff, Student satisfaction with services provided, The average student satisfaction)
99. **Graduates skills, Graduates qualification**,
100. **Administration and Finance** (Annual budget, Research budget according to the source of funding, University revenue, Annual cost per student,)
101. **Human Resources** (Faculty by qualification, Faculty by gender, International component of Faculty, The Faculty pyramid-professor-associate-assistance, Teaching staff at the Centre for Preparatory Studies, Administrative and support staff by qualification and gender, Administrative and support staff by qualification and nationality, Technical staff by qualification and gender, Technical staff by qualification and nationality, Number of students per staff member, Number of students per lecturer in the Foundation Programme, Comparison of number of students per staff member by category for the Present and the Previous Periods, Staff on scholarship, Staff who benefited from training programs within and outside the Sultanate, Staff turnover,) ([9](#_ENREF_9))
102. **Enrollment** (College enrollment is tracked by term and by campus.).
103. **Retention Rate**
104. **Graduation Rate**
105. **Course Success Rate**
106. **Achievement of Program Graduates:** Performance on Licensing Examinations
107. **National Educational Examinations (**[**10**](#_ENREF_10)**).**
108. **Incoming Freshmen**
109. **Enrollment**
110. **New undergraduate student’s enrollment**
111. **Full time, first year students receiving VAL & SAL scholarships**
112. **Online enrollment**
113. **NSSE high impact practices**
114. **High impact practices participation comparison** (Percentage of students who participated in High-Impact Practices)
115. **How many of your courses at this institution have included a community based project (service learning)?**
116. **Participate in a learning community or some other formal program where groups of students take two or more classes together**
117. **Development contribution by fund**
118. **Development contribution by constituency** ([11](#_ENREF_11)).
119. **The opinions that were obtained by asking the teaching staff about their views of the mission and to what extent it fits the expectations and the society needs**
120. **The awareness and support of the program teaching staff and the administrative staff of its mission**
121. **Evaluating the effectiveness of governance and leadership**
122. **Evaluating the general performance of administration**
123. **The extent of awareness of the department teaching staff, the students and the administrative staff of the existence of general regulations and policies concerning and affecting them and how well they know these regulations**
124. **The proportion of courses in which student evaluations were conducted during the year**
125. **The extent of the existence of quality improvement plans including indicators, benchmarks on the program level.**
126. **Proportion of programs in which there was independent verification within the institution of standards of student achievement by internal/external processes during the past year.**
127. **Ratio of students to teaching staff** (based on full time equivalents) in the program/ the college/ the university to the number of the teaching staff in the program/ the faculty/ the university.
128. **Student overall rating on the quality of their courses. (**Average rating by students on a five point scale on overall evaluation of courses.)
129. **Proportion of teaching staff with verified doctoral qualifications.**
130. **Proportion of students entering undergraduate programs who complete those programs in minimum time.**
131. **The appropriateness of the teaching methods according to student evaluation, external reviewers and the teaching staff for each of the learning domains as attained in the NQF.**
132. **Proportion of graduates from undergraduate program who within 6 months of graduation are: a. Employed b. enrolled in further study c. not seeking employment or further study**
133. **Students’ evaluation of the value and quality of field activities**
134. **The evaluation of the quality and usefulness of the courses by advisory bodies of the industrial and professional sectors and other distinctive community sectors.**
135. **The appropriateness of the qualifications and experience of the teaching staff for the courses they teach**
136. **Student evaluation of the academic and career counseling.**
137. **Students enrolled in graduate programs ratio**
138. **The student’s services financial stake according to the total operational expenses**
139. **The number of cases in which a disciplinary action was taken (**Number of cases (Students irregularities such as the absence, cheating, misbehave) in which a disciplinary action was taken such as Academic warning, Deprivation, Suspensions, enrollment collapse, elimination according to the total number of regular students 100%. (
140. **The percentage of the students who participate with non - classes activities**
141. **Ratio of students to administrator’s staff (Institutional only).**
142. **Number of books and magazines and total number of brochures for each regular student (Books ratio to students)**
143. **Number of Magazines participations according to the available programs numbers**
144. **Expenses average for books and magazines per each student.**
145. **Student's evaluation for the libraries services (average rating on adequacy services on a five points scale for senior student’s annual survey.**
146. **The number of databases retina available to students and faculty members through the library.**
147. **The allocated financial spending ratio on the education materials and equipment's.**
148. **Average number of computers available for each student's use.**
149. **Malfunctions repair expenses.**
150. **Information technology expenses average per student**
151. **Opinion reconnaissance about the users’ satisfaction for use of the class rooms, laboratories', information devices unites**
152. **Student average cost**
153. **The income ratio the comes from different resources (governmental, research, etc.)**
154. **Observations, external financial auditing reports**. (To identify the financial reports quality ratio, through the auditing from external auditors (
155. **Percentage of faculty members who have left the work in the organization during the last academic year for reasons other than the contract**
156. **The number of formal complaints issued from faculty and administration staff compared to the total number of complaints and disputes.**
157. **Ratio of Ph.D. or equivalent degree teaching faculty members ratio according to other regular faculty members**
158. **Ratio of foreigners' faculty members**
159. **Average ratio of the teaching staff members’ evaluation in the program for the adequacy of scientific research facilities and equipment**
160. Number of scientific researches published in the scientific refereed journals in the previous year for each teaching staff member working for full-time or equivalent
161. **Number of research and innovations registered as an intellectual property and patents within the past five years**
162. **The number of citations in scientific refereed journals of all teaching staff members researches during the last academic year.**
163. **The percentage of students enrolled in graduate programs that have completed the requirements of these programs on time.**
164. **The number of scientific paper presented in the conferences on the national or international level proportion to the number of full-time teaching staff members**
165. **The percentage of the total expense of the university on the research**
166. **Assessment the level of satisfaction of employers / companies / service users / Alumni Association / parents / about the graduate’s efficiency**
167. **The percentage of full-time teaching staff members and the others of administrative staff that participate in community services activities.**
168. **The number of community services programs proportion to the number of scientific departments (**[**12**](#_ENREF_12)**).**
169. **student satisfaction and engagement**
170. **graduate satisfaction**
171. **employer satisfaction**
172. **graduate employment rate**
173. **graduation rate**
174. **Apprenticeship satisfaction and engagement** ([13](#_ENREF_13))
175. **Awards Conferred**
176. **Enrollment**
177. **Retention Rate**
178. **Transfer-out Rate**
179. **Graduation Rate** ([14](#_ENREF_14))
180. **State Performance Funding Measures**
181. **Enrollment** (Enrollment by Specified Group, Enrollment by Diversity, Enrollment by Citizenship, Enrollment by Geographic Origin)
182. **Student Success** (First-time Full-time New in College (FTFTNIC) Students Completing 24 Credit Hours in Their First Year, First to Secondary Year Retention Rate, Four-Year Graduation Rate, Six-Year Graduation Rate, Knowledge Rate, Annual Success ratio, Employed part-time, Employed full-time, Not seeking employment/Continuing Ed, Seeking employment, Planning to continue Ed (not enrolled), Enrolled in continuing Ed program, Volunteer/Service Program)
183. **Quality of Student Learning (University Exit Exam)**
184. **Graduate Outcome (Degrees Awarded)**
185. **Faculty and Staff** (Student to Faculty Ratio, New Faculty Hired by Academic Year, Faculty by Rank, Faculty by Race/Ethnicity, Underrepresented Faculty by Race/Ethnicity, Faculty Scholarship, Faculty Salaries, Staff by Race/Ethnicity, Percent of Staff Salaries at or Above the College and University Professional Association Median, Underrepresented Staff by Race/Ethnicity)
186. **Sustainability and Financial Efficiency** (The Association for the Advancement of Sustainability in Higher Education Evidence, Buildings, Grounds, Land, Academic and Administrative Space per full time equivalent, Energy Use Index)
187. **Funding** (Undergraduate Cost of Attendance, Graduate College Cost of Attendance, Distribution of Total Expenditures to the Core Mission, Faculty/Staff Grant Submissions, Annual Gift Commitments) ([15](#_ENREF_15)).
188. **NSS and internal survey ratings**
189. **Graduate employability**
190. **Proportion of good degrees**
191. **Student retention rates**
192. **Student entry tariffs**
193. **National teaching awards**
194. **Student Union National Student Survey ratings**
195. **Volume of SU activities** (societies, events, etc)
196. **Organization and management score in National Student Survey**
197. **Student satisfaction with campus environment**
198. **% of Estate in condition A/B**
199. **% of first year students housed on campus**
200. **Proportion of academic staff returned in the REF**
201. **REF ratings by academic return**
202. **External research grant income**
203. **Number of full-time PGR students and their completion rates**
204. **Grant income from industry and commerce**
205. **Quality of REF Impact case studies**
206. **Number of active UK strategic partners** (including schools, trusts and hospitals)
207. **Volume of CPD work delivered**
208. **External footfall on campus, including to Sporting Edge and the Arts Centre**
209. **The number of KTPs and other enterprise partnerships with external businesses**
210. **Size of active alumni base**
211. **Proportion of total tuition fee income from overseas students**
212. **Percentage of OS students at Edge Hill campus**
213. **Number of student registrations at OS campuses**
214. **Number of international research collaborations**
215. **Proportion of non-UK academic staff**
216. **Number of home students studying abroad**
217. **Staff turnover**
218. **Outcome of staff survey**
219. **Participation in performance review and development**
220. **Equal pay audit**
221. **Sickness absence**
222. **Ratio of applications to places**
223. **Conversion rates**
224. **Scale of retained surplus**
225. **Levels of borrowing**
226. **Proportion of staff costs**
227. **Cash balances (**[**16**](#_ENREF_16)**)**
228. **Graduation rates**
229. **Course Completion rates**
230. **Satisfaction**
231. **Retention rates**
232. **Learner Demographics**
233. **Employment (**[**17**](#_ENREF_17)**)**
234. **Course Success KPIs** (college-level course success, online course success, developmental course success)
235. **Retention KPIs** (fall to spring retention full time students, fall to spring retention part time students, fall to fall retention full time students, The Integrated Postsecondary Education Data System (IPEDS) Peer Comparison fall to fall retention first time full time, fall to fall retention part time students, IPEDS fall to fall retention Peer Comparison first time part time)
236. **Credit Momentum KPIs** (full time students earning 12 college level credits in first year, part time students earning 6 college level credits in first year, full time students earning 30 college level credits in first year, full time students earning 24 college level credits in first year, part time students earning 15 college level credits in first year)
237. **Graduation and Transfer KPIs** (IPEDS 3-year graduation rate, IPEDS 3 year transfer rate, National Community College Benchmark Project (NCCBP) 3-year graduation rate full time, NCCBP 3-year graduation plus transfer rate full time, NCCBP 6-year graduation rate part time, NCCBP 6 year graduation rate full time, NCCBP 6 year graduation plus transfer rate part time, NCCBP 6 year graduation plus transfer rate full time, year 2020 graduation goal)
238. **Enrollment KPIs** (fiscal year The Financial Times Stock Exchange (FTSE) trend, fall headcount trend)
239. **Student Learning Outcomes KPIs** (adjunct faculty participation rate in Medical Counseling Committee (MCC) 4Cs, residential faculty participation rate in MCC 4Cs) ([18](#_ENREF_18))
240. **% students participating in high-impact practices**
241. **Level of student engagement** (NSSE scores; senior survey)
242. **% students with experience record**
243. **Post-graduation placement rate**
244. **Student loan repayment rate**
245. **On-time (four-year) graduation rate**
246. **Total bachelor’s degrees conferred**
247. **Online degrees conferred**
248. **Certificates, micro-credentials, alternative credentials awarded**
249. **Degrees and alternative credentials awarded to alumni**
250. **# of degrees/alternative credentials awarded in high impact fields**
251. **# of formal educational partnerships with Indiana employers/partners**
252. **# of graduate degree recipients employed in Indiana**
253. **% of faculty/staff/students involved in community-engaged activities**
254. **# of formal community engagement programs/projects in East Central Indiana**
255. **Community and industry partner satisfaction surveys**
256. **Community Vitality Indicators**: (Quality of Life, Economic Vitality, Sustainability, Educated Citizenry)
257. **# local and regional scholarship projects**
258. **# scholars/projects receiving state/national/international recognition**
259. **# of students involved in undergraduate research**
260. **Total external grants and contracts expenditures**
261. **Research grants and contracts expenditures**
262. **Composite financial index**
263. **level of annual donor commitments**
264. **Student affordability/Net price**
265. **Carbon neutrality**
266. **Employee satisfaction/engagement/well-being survey**
267. **% faculty/staff diversity**
268. **Campus climate survey (**[**19**](#_ENREF_19)**)**
269. **Student Demand and Quality:** (Curtin market share of WA university students – total commencements, Tertiary Institutions Service Centre (TISC) first preferences and commencements with ATAR ≥95)
270. Student Experience: (Student satisfaction (SES) – undergraduate students, Retention rate – undergraduate students)
271. Graduate Outcomes: (Domestic graduate employment rates)
272. Student Equity: (Percentage of total domestic enrolments – regional and remote students, Total enrolments – Aboriginal and Torres Strait Islander students)
273. Research Performance: (Publications per Research Only/Teaching and Research staff FTE, Total research income (Category 1 – 4), Completion numbers – Higher Degree by Research (HDR) students)
274. Industry Engagement: (Category 3 and 4 industry income and industry scholarships)
275. Staff Engagement: (Staff engagement survey results)
276. Diversity and Equity: (Number of Aboriginal and Torres Strait Islander staff and internships, Staff gender balance)
277. International Reputation: (International co-authorship, ARWU ranking)
278. Financial Sustainability: (Teaching and Learning (T&L) expenditure per EFTSL, Revenue from non-Commonwealth sources) ([20](#_ENREF_20))

**References:**

1. Lakehead University. 2016 Performance Indicators. Ontario, Canada: Lakehead University, 2016.

2. Ontario C. 2018-19 key performance indicators. Ontario, Canada: Colleges Ontario, 2019.

3. University of Arkansas. University of Arkansas at Monticello Academic Unit Annual Report. Arkansas, USA: School of Nursing (SON), 2019.

4. Office of Planning & Analysis. Academic Key Performance Indicators (KPIs) Montana, USA: Montana State University, 2017.

5. Education Evaluation Commission. Nursing Program Specification. Saudi Arabia: ALJOUF university College of Applied Medical Sciences, Department of nursing, Al-Qurayyat., 2018.

6. Hegazi M. College of nursing strategic plan 2015-2020. Dammam, Saudi Arabia: University of Dammam, 2016.

7. Chicago State University. Program Assessment. Chicago, USA: Department of nursing, 2021.

8. Office of Institutional Analysis. Ministry of Colleges and Universities Key Performance Indicators. Windsor, Canada: University of Windsor, 2020.

9. Planning & Statistics Department. Key performance Indicators Third Issue (2016-2013). Muscat, Oman: Sultan Qaboos University.

10. Gadsden State Community College. Key Performance Indicators (KPIs) 2017-2018. United States: Gadsden State Community College, 2018.

11. University of the Virgin Islands Planning Committee. key performance indicators. University of the Virgin Islands, 2020.

12. Quality and Development Department. Key Performance Indicators of the University of Hail (Definition-Patterns- Importance). Kingdom of Saudi Arabia.

13. Georgian College. Key Performance Indicators Ontario, Canada: Georgian College; 2021 [25/April/2021]. Available from: https://[www.georgiancollege.ca/about-georgian/corporate-information/key-performance-indicators/](http://www.georgiancollege.ca/about-georgian/corporate-information/key-performance-indicators/).

14. Operational Departments. Key Performance Indicators. Illinois, United States: Prairie State College, 2018.

15. Office of Institutional Research. Key Performance Indicators. Missouri, United States: Missouri State University, 2021.

16. Edge Hill University. Key Performance Indicators 2020 [updated 22/September/202013/April/2021]. Available from: https://[www.edgehill.ac.uk/corporate-information/board-of-governors/resources/section-b/key-performance-indicators/](http://www.edgehill.ac.uk/corporate-information/board-of-governors/resources/section-b/key-performance-indicators/).

17. California Southern University. Key Performance Indicators California, United States [28/April/2021]. Available from: https://[www.calsouthern.edu/key-performance-indicators/](http://www.calsouthern.edu/key-performance-indicators/).

18. MCC Office of Institutional Effectiveness. MCC Key Performance Indicators 2017-18 Arizona, United States: Mesa Community College.

19. Ball State University. executive-dashboard United States: Ball State University; 2019 [18/April/2022]. Available from: https://[www.bsu.edu/about/strategic-plan/executive-dashboard#accordion_question10](http://www.bsu.edu/about/strategic-plan/executive-dashboard#accordion_question10).

20. Annual Report 2020. Australia: Curtin University, 2021.
