# Supplementary material for: Baccalaureate nursing education institutions’ key performance indicators: a review of the existing indicators and qualitative analysis of expert interviews
Source: BMC Nurs. 2023 Oct 5;22:357. doi: 10.1186/s12912-023-01484-6 (PMC10552274; doi:10.1186/s12912-023-01484-6)
Supplement: Supplementary file 2 — Supplementary Material 2 [file 12912_2023_1484_MOESM2_ESM.docx]

**Indicators explanation:**

In this study, we reported indicators that can be considered by research groups who are intended to design a set of indicators related to nursing education institution performances. Many details related to indicators like indicators definition, interpretation, and reporting of the indicators, detailed metadata describing each indicator and details of computation, guidance on the data sources, the minimum data set that needs to be collected, and frequency of data collection should be considered when you are using and reporting the indicators while the target audience is kept in mind and the use for the measurement is specified. Here we brought the selected indicators in the study and a brief explanation about some of the indicators to make them more clear.

Table S1: Selected indicators and a brief explanation for some indicators.

| **Indicators** | | **Explanation** |
| --- | --- | --- |
| 1 | Ratio of students to administrators’ staff | - |
| 2 | Ratio of students to teaching staff. | - |
| 3 | Staff by qualification and gender. | - |
| 4 | Tenure track faculty headcount. | The number of assistant, associate, and full professors working in a school at any given time. |
| 5 | Indigenous staff participation rate. | - |
| 6 | The proportion of faculty members with more than two years of clinical experience. | - |
| 7 | The proportion of faculty members with expertise in clinical management. | - |
| 8 | The proportion of faculty members with executive experience outside of college. | - |
| 9 | The proportion of teaching staff participating in professional development activities. | It means participating in various activities that can help teaching staff improve their professional skills, knowledge, and ability, such as continuous education |
| 10 | Staff turnover. | - |
| 11 | The average number of computers available for each student's use. | - |
| 12 | The number of databases retina available to students and faculty members through the library. | - |
| 13 | The quality of the digital library. | - |
| 14 | The number of online full-text dissertations. | - |
| 15 | Books ratio to students. | - |
| 16 | The overall quality of the facilities/resources in the college. | - |
| 17 | The overall quality of the services in the college. | - |
| 18 | The number of active strategic partners, including schools trusts and hospitals. | - |
| 19 | Buildings, Grounds, Land, Academic and Administrative Space per full-time equivalent. | - |
| 20 | Total grants received. | - |
| 21 | Institutional scholarship total amount. | - |
| 22 | Total annual donor commitments. |  |
| 23 | The income ratio that comes from different resources. | - |
| 24 | Annual budget. | - |
| 25 | Funding. | - |
| 26 | The extent of awareness of the department teaching staff, the students, and the administrative staff of the existence of general regulations and policies concerning and affecting them. | - |
| 27 | Stakeholder evaluation of the Policy Handbook, including administrative flow chart and job responsibilities | - |
| 28 | Teaching effectiveness-student evaluation. | This indicator refers to the results of students' evaluation of the effectiveness of teaching |
| 29 | Teaching effectiveness-chairman evaluation. | This indicator refers to the results of chairman' evaluation of the effectiveness of teaching |
| 30 | Teaching effectiveness-peer evaluation. | This indicator refers to the results of peer' evaluation of the effectiveness of teaching |
| 31 | Students’ overall rating on the quality of their courses. | - |
| 32 | Students’ evaluation of the value and quality of field activities. | - |
| 33 | The appropriateness of the teaching methods according to student evaluation, external reviewers, and the teaching staff for each of the learning domains. | - |
| 34 | The appropriateness of the qualifications and experience of the teaching staff for the courses they teach. | - |
| 35 | Quality of online teaching. | - |
| 36 | Online Student Credit Hour. | - |
| 37 | The volume of continuing professional development work delivered. | - |
| 38 | Level of student engagement. | The level of student participation in the learning process |
| 39 | Weekly Student Contact Hours. | The number of hours that teaching staff allocates to students in a planned way every week, including classrooms, online counseling, etc. |
| 40 | The number of periodic and regular meetings between faculty members, staff, and managers. | - |
| 41 | Membership in international organizations. | - |
| 42 | International activities by faculty members per college. | - |
| 43 | The number of international research collaborations. | - |
| 44 | Number of faculty engaged in professional organizations. | - |
| 45 | The number of membership of boards and committees outside the University. | - |
| 46 | The number of knowledge transfer partnerships and other enterprise partnerships with external businesses. | - |
| 47 | The proportion of full-time teaching and other staff actively engaged in community service activities. | - |
| 48 | High impact practices participation. | Students’ engagement in activities that promote deep learning |
| 49 | Stakeholder evaluation of Overall: a) Websites, b) e-learning services, c) Hardware and software, d) Accessibility e) Learning and Teaching, f) Assessment and service, g) Web-based electronic data management system or electronic resources. | - |
| 50 | The evaluation of the quality and usefulness of the courses by advisory bodies of the industrial and professional sectors and other distinctive community sectors. | - |
| 51 | Evaluating the effectiveness of governance and leadership. | - |
| 52 | Evaluating the general performance of administration. | - |
| 53 | Student evaluation of academic and career counseling. | - |
| 54 | Campus climate survey. | Participants’ perception of school climate |
| 55 | National student survey and internal survey ratings. | - |
| 56 | Outcome of alumni survey. | - |
| 57 | The outcome of staff survey. | - |
| 58 | Evidence of current accreditation by a national nursing accrediting body. | - |
| 59 | Number of faculty/student awards granted. | - |
| 60 | School international ranking. | - |
| 61 | School national ranking. | - |
| 62 | Total webpage visits along with new visitors. | - |
| 63 | Top-of-Mind Awareness. | That the name of the school is the first name that comes to mind among the names of the schools |
| 64 | Web Analytics. | Measuring data related to web usage. |
| 65 | Earned Media. | It means the contents that are produced about your institution without you paying for it. |
| 66 | Student satisfaction rate. | - |
| 67 | Graduate satisfaction rate. | - |
| 68 | Staff satisfaction rate. | - |
| 69 | Patient satisfaction rate. | - |
| 70 | Patients’ family satisfaction rate. | - |
| 71 | Employer Satisfaction rate. | - |
| 72 | Energy Use Index. | Energy used per square foot or meter per year |
| 73 | Carbon neutrality. | Net zero carbon dioxide emissions. Having a balance between emitting carbon and absorbing carbon. |
| 74 | The Association for the Advancement of Sustainability in Higher Education Evidence. | AASHE is the leading association for the advancement of sustainability in higher education. |
| 75 | Composite Financial Index. | It is a composite indicator that shows the university’s financial health and is consisted of four indicators: Primary Reserve Ratio, Viability Ratio, Return on Net Assets Ratio, and Net Operating Revenues Ratio |
| 76 | Student Loan Repayment Rate. | - |
| 77 | Annual cost per student. | - |
| 78 | Total teaching & learning expenditure. | - |
| 79 | Total operating expenditure. | - |
| 80 | The student’s services financial stake according to the total operational expenses. | - |
| 81 | Total expense of the university on the research. | - |
| 82 | Faculty Instructional Full Time Equivalent paid in program. | - |
| 83 | Enrollment rate. | - |
| 84 | Student entry tariffs score. | The tariff is designed to make different types of qualifications comparable and also to enable comparability between students. |
| 85 | The student’s cumulative grade point average (CGPA). | It can be calculated by dividing the sum of the grade points earned by the total credit value of courses. |
| 86 | Health Education Systems Incorporated (HESI) exit examination. | It can be any standard exit examination that takes place in nursing schools around the world. |
| 87 | National Educational Examinations passing rates. | - |
| 88 | Average number of semesters a student spends under probation. | - |
| 89 | College-level course success rate. | - |
| 90 | Total number of online degrees conferred. | - |
| 91 | Total bachelor’s degrees conferred. | - |
| 92 | Transfer-in rate. | It means students who have transferred to this institution. |
| 93 | Transfer-out rate. | It means students who have transferred from this institution. |
| 94 | Retention rate. | - |
| 95 | Graduation rates. | - |
| 96 | On-Time graduation rate. | - |
| 97 | Assessment of Student Learning: student exit survey. | The purpose of the indicator is to examine the students' learning based on the survey of the students themselves. |
| 98 | Assessment of Student Learning: alumni and employers' surveys. | The purpose of the indicator is to examine the students' learning based on the survey of the alumni and employers. |
| 99 | Level of Interest in the field . | To indicate to what extent the institution has been successful in creating interest in the field of nursing. |
| 100 | Graduates assessment results (Cognitive, Function, Attitude, Problem Solving, communication, decision making, management, planning, familiarity with legal issues, social responsibility, altruism, self-confidence, professionalization, professional dignity, and ethics). | These are characteristics that, according to the participants, are needed in the nursing graduates of an institution to represent the institution's performance. |
| 101 | Proportion of graduates from undergraduate program who enrolled in further study. | - |
| 102 | Passing Rates for Licensure Exams (like NCLEX-RN). | - |
| 103 | Employment rate. | - |
| 104 | Percentage of immigration and finding related jobs abroad. | - |
| 105 | Quality of provided care by alumni. | - |
| 106 | Nurse-sensitive indicators. | These indicators are affected by nursing care and can show the quality of nursing care. |
| 107 | The number of publications in the form of books, book chapters, and technical reports. | - |
| 108 | The number of scientific papers presented in the conferences on the national or international level. | - |
| 109 | The number of research and innovations registered as intellectual property and patents. | - |
| 110 | The number of scientific researches published in the scientific refereed journals. | - |
| 111 | The number of citations in scientific refereed journals of all teaching staff members’ research | - |
| 112 | The number of nursing models developed. | It means models that are the result of research done in the school by students and staff |
